# Scrapie Agent (Strain 263K) Can Transmit Disease via the Oral Route after Persistence in Soil over Years

**DOI:** 10.1371/journal.pone.0000435

**Published:** 2007-05-09

**Authors:** Bjoern Seidel, Achim Thomzig, Anne Buschmann, Martin H. Groschup, Rainer Peters, Michael Beekes, Konstantin Terytze

**Affiliations:** 1 Fraunhofer Institute for Molecular Biology und Applied Ecology (IME), Schmallenberg, Germany; 2 P24 -Transmissible Spongiform Encephalopathies, Robert Koch-Institut, Berlin, Germany; 3 Institute for Novel and Emerging Infectious Diseases, Friedrich-Loeffler-Institut, Insel Riems, Germany; 4 German Federal Environmental Agency (Umweltbundesamt, UBA), Dessau, Germany; Massachusetts General Hospital & Harvard Medical School, United States of America

## Abstract

The persistence of infectious biomolecules in soil constitutes a substantial challenge. This holds particularly true with respect to prions, the causative agents of transmissible spongiform encephalopathies (TSEs) such as scrapie, bovine spongiform encephalopathy (BSE), or chronic wasting disease (CWD). Various studies have indicated that prions are able to persist in soil for years without losing their pathogenic activity. Dissemination of prions into the environment can occur from several sources, e.g., infectious placenta or amniotic fluid of sheep. Furthermore, environmental contamination by saliva, excrements or non-sterilized agricultural organic fertilizer is conceivable. Natural transmission of scrapie in the field seems to occur via the alimentary tract in the majority of cases, and scrapie-free sheep flocks can become infected on pastures where outbreaks of scrapie had been observed before. These findings point to a sustained contagion in the environment, and notably the soil. By using outdoor lysimeters, we simulated a contamination of standard soil with hamster-adapted 263K scrapie prions, and analyzed the presence and biological activity of the soil-associated PrP^Sc^ and infectivity by Western blotting and hamster bioassay, respectively. Our results showed that 263K scrapie agent can persist in soil at least over 29 months. Strikingly, not only the contaminated soil itself retained high levels of infectivity, as evidenced by oral administration to Syrian hamsters, but also feeding of aqueous soil extracts was able to induce disease in the reporter animals. We could also demonstrate that PrP^Sc^ in soil, extracted after 21 months, provides a catalytically active seed in the protein misfolding cyclic amplification (PMCA) reaction. PMCA opens therefore a perspective for considerably improving the detectability of prions in soil samples from the field.

## Introduction

Transmissible spongiform encephalopathies (TSEs) comprise a group of fatal neurodegenerative diseases such as bovine spongiform encephalopathy (BSE) in cows [1], chronic wasting disease (CWD) in deer (*Odocoileus* spp.) and elk (*Cervus elaphus nelsoni*) [2]–[4], scrapie in sheep and goats [5]–[7] and Creutzfeldt-Jakob disease (CJD) in humans [1]. The exact molecular pathomechanisms underlying TSEs have not yet been fully elucidated but it is generally accepted that a pathologically misfolded and/or aggregated isoform of the normal cellular prion protein (PrP), referred to as PrP^Sc^ and PrP^C^, respectively, is the key pathogenic factor for this group of diseases [1].

Among the known TSEs, only scrapie and CWD are contagious diseases which show horizontal transmissibility under natural conditions [2], [4], [8]. CWD is the only TSE known to affect free-ranging animals [3]. The regular occurrence of scrapie in affected areas [7] and the spread of CWD in North America and Korea [9], [10] among mule deer, white-tailed deer and elk indicates that a contagion in the environment is responsible for the occurrence of these TSEs [4], [11]–[14], and even raises the possibility of a cross-species transmission under natural conditions. Recent findings demonstrated that saliva from deer with CWD harbours infectivity and can transmit this TSE upon peroral uptake [15]. Other studies pointed to transmission of scrapie among sheep by vectors like mites, fly larvae or other ectoparasites [16]–[19]. It has also been hypothesized that sporadically occurring TSEs may be induced by insecticides or by a disproportion of manganese and copper in soil leading to an enrichment of manganese in animals [20], [21]. Alternatively, an influence of the Fe/Mn ratio in forage has been discussed in connection with TSEs [22]. However, on the balance of all evidence available so far, contaminated soil appears as one of the most likely sources of infection in the natural transmission of scrapie and possibly also CWD. It has been known for decades that sheep can become infected with scrapie while grazing on pastures where infected sheep have been kept before, and scrapie occurs often in areas where it has already occurred previously [7], [8], [23]. Furthermore, Brown and Gajdusek found that scrapie agent remains infectious after persisting in soil for 3 years as evidenced by intracerebral bioassay in Syrian hamsters [24]. The putative transmission of scrapie and CWD via soil is also corroborated by recent studies showing that prion infectivity binds to soil components with high affinity [25], [26], thereby retaining its pathogenic biological activity [25]. Thus, soil-associated TSE agents in the environment represent a potential hazard. This holds true not least since prion infectivity exhibits an unusually pronounced resistance against both physical and chemical methods of inactivation, as described in detail elsewhere [27]–[37].

The contamination of soil with TSE infectivity can occur from several sources. Since recent studies could demonstrate that scrapie infectivity is present in various tissues and body fluids of infected animals [38], [39], it has been assumed that the persistent prion protein enters the environment by contaminated excrements, birth-related tissues such as placenta, or even whole carcasses. While a similar excretion pattern appears conceivable for the CWD agent [40] which has also been found in saliva [15], the present knowledge about the BSE pathology in cattle does not argue for a significant shedding of the infectivity via faeces, urine, or during birth [41], [42]. However, small ruminants infected with BSE could supposedly spread the BSE agent throughout the environment in a similar manner as known from scrapie-affected sheep or CWD-affected animals [43]. Moreover, unconventional conditions like for example the burial of animal carcasses at larger numbers as practiced for example during the foot and mouth disease outbreak in the UK in 2001 [44], may have fostered a dissemination of BSE agent in the soil or ground water. Once deposited there, all three TSE agents, BSE, scrapie, and CWD, must be assumed to persist in an infectious state for long periods of time.

So far, the oral transmission efficacy of long-term prion contaminations in soil have not been investigated. Therefore, we have studied the persistence of PrP^Sc^ in the environment over time and measured its oral transmissibility by bioassay in Syrian hamsters. With outdoor lysimeter experiments we simulated the situation on pastures using soil spiked with scrapie-infected hamster brain homogenate over a period of 29 months and analyzed the fate of the prion proteins by sensitive Western blotting and, in part, also by protein misfolding cyclic amplification (PMCA). The infectivity of such contaminated soil samples and the respective aqueous soil extracts was tested in the hamster bioassay.

## Results

### Extraction and Recovery

**Figure 1:**
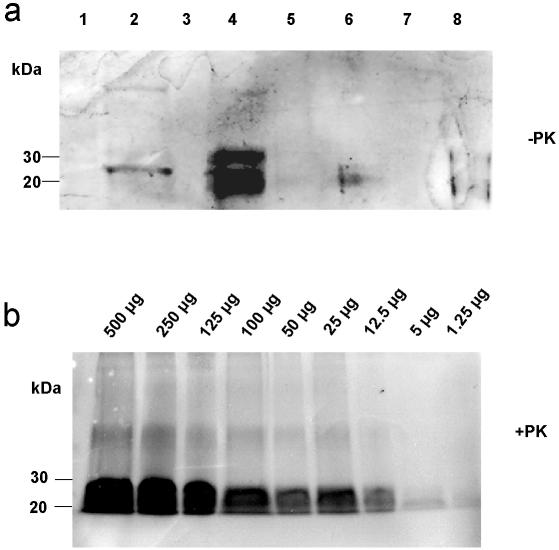
Western blot analysis of short-time incubation experiments. a) Western blot detection of PrP^C^ extracted from soil mixed with non-infectious brain homogenate (5% pork brain in German standard soil). Several different buffers and solutions were used for extraction. Lane 1: water; lane 2: Triton X-100; lane 3: 1% urea; lane 4: 1% SDS; lane 5: Zwittergent; lane 6: RIPA buffer; lane 7: NP-40; lane 8: Na-sarcosyl. b) Western blot detection of PrP27-30, the proteinase K-resistant core of PrP^Sc^, extracted by using 1% SDS from soil contaminated with 263 K scrapie brain homogenate from hamsters after 1 h of incubation (dilution series). PrP^Sc^ could be detected in soil samples containing 1.25 µg or higher amounts of scrapie brain tissue after extraction with 1% SDS-solution. Samples were digested with proteinase K prior to Western blotting.

In the first phase of the study several buffers – known as standard buffers for protein extraction from mammalian cells – and other solutions were tested in order to find out the optimal extraction method for prion protein from contaminated soil (see Materials and Methods). The obtained results lead us to use 1% SDS (sodium dodecyl sulphate) in sterilized water (figure 1a), which allowed detection of PrP^Sc^ in samples of German standard soil containing an amount of PrP^Sc^ that corresponded to 1.25 µg of infectious brain material (figure 1b).

### Soil Incubation Experiments

**Figure 2:**
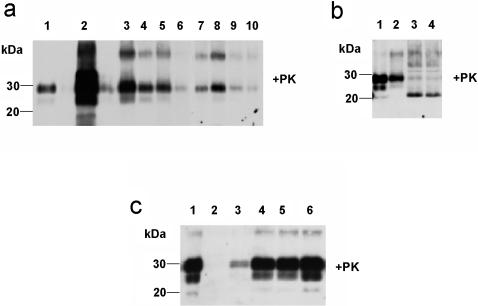
Western blot analysis of long-time incubation experiments. a) Western blot detection of PrP27-30 extracted from prion-contaminated soil after different time periods. Lane 1: PK-digested 263K scrapie hamster brain homogenate containing 5×10^−7^ g of brain tissue (positive control); lanes 2–10: PrP27-30 extracted at time point 0 (lane 2), after 1 month (lane 3), after 3 months (lane 4), after 6 months (lane 5), after 12 months (lane 6), after 18 months (lane 7), after 21 months (lane 8), after 26 months (lane 9) and after 29 months (lane 10). b) Deglycosylated PrP27-30 extracted from prion contaminated soil. Lane 1: PK-digested 263K scrapie hamster brain homogenate containing 5×10^−7^ g of brain tissue (positive control); lane 2: soil-extracted PrP27-30 after 21 months; lanes 3 and 4: deglycosylated soil-extracted PrP27-30 after 21 months (lane 3) and after 18 months (lane 4). c) PMCA amplification of PrP^Sc^ extracted from contaminated soil. Lane 1: PK-digested 263K scrapie hamster brain homogenate containing 5×10^−7^ g of brain tissue (positive control); lanes 2–6: sample signals after 0, 40, 80, 120 and 160 cycles of PMCA, respectively.

The findings from our lysimeter experiments indicated a remarkable persistence of PrP^Sc^ in soil by clearly showing that - even after an incubation for 29 months - PrP***^Sc^*** could be still extracted from soil and detected by Western blotting. These results were confirmed by using two alternative highly sensitive Western blotting techniques. As shown in figure 2a, PrP***^Sc^***, in the form of its protease-resistant core of the pathological prion protein PrP^Sc^ (PrP27-30), is still detectable in soil after 29 months. However, a decrease in the extractable concentration is obvious. The strongest decrease can be seen during the first month of incubation in the soil (figure 2a, lane 2 and 3). After three months the extractable amount of PrP27-30 remained almost constant until month 21, with small variations at month 12 and month 18 (figure 2a, lane 6 and 7). In Fig. 2a, a slightly stronger signal for PrP27-30 was found for 21 months (lane 8) as compared to 18 months (lane 7). Both samples were taken from the same soil bag, however, the location from where the sampling was performed may have differed. Thus, the finding possibly reflects minor inhomogenities either in the original load of contamination or in the degradation of PrP^Sc^ depending on the microenvironment of the examined sample. On the other hand, it has to be noted that the extraction yield and Western blotting efficiency inevitably may also vary in certain ranges from sample to sample. A further decrease was observed after 26 and 29 months (figure 1a, lane 9 and 10), however, the PrP27-30 specific bands remain clearly visible. Where performed, analyses of samples from different soil bags produced consistent results. In order to verify that the detected bands originated from PrP^Sc^ a control experiment was performed: After deglycosylation with PNGase F the PrP27-30 bands extracted from soil after 21 and 18 month showed an electrophoretic shift towards a single band at about 20 kDa, the molecular mass to be expected for deglycosylated PrP27-30 from 263K hamster scrapie (figures 2 b, lanes 3 and 4, respectively).

### Amplification of PrP^Sc^ extracted from soil

In order to check whether PrP^Sc^ extracted from soil can be used for the amplification of protease resistant prion protein by the protein misfolding cyclic amplification (PMCA) reaction [45], [46], soil extracts from month 21 were used as test seeds. As shown in figure 2c, a strong increase of the signal for PrP^Sc^ was obtained with this method: while no signal for PrP^Sc^ can be detected in samples without PMCA treatment (lane 2), clear signals became visible after 40 cycles of PMCA (lane 3) and even more intense after 80, 120 and 160 cycles of PMCA (lanes 4, 5 and 6, respectively).

### Presence of PrP^Sc^ in surrounding soil samples

**Figure 3:**
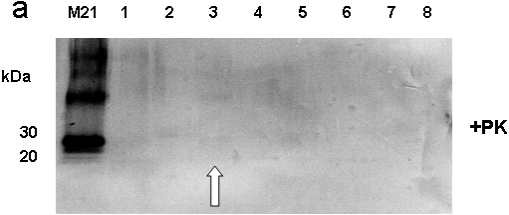
Western blot detection of PrP27-30 extracted from prion-contaminated soil after 21 months (M21) and respective surrounding samples. M21: contaminated soil sample inside the gauze bag; lane 1: soil sample collected outside of the steel cage; lane 2: soil sample collected directly over the gauze bag; lane 3: analysis of the empty gauze bag; lane 4: soil sample collected underneath the steel cage; lane 5: soil sample collected directly next to the gauze bag; lane 6: roots collected next to the gauze bag; lane 7: soil sample collected underneath the gauze bag; lane 8: non-contaminated soil. Arrow head at lane 3 indicates a faint PrP27-30 signal, resulting from residual soil particles that remained attached to the gauze bag.

For analyzing the fate of the prion protein in more detail, soil samples in the vicinity of the gauze bag, as well as the gauze bag itself were analyzed by Western blotting. As shown in figure 3a, no PrP^Sc^ specific signal could be detected in the surrounding soil samples. This is indicative of PrP***^Sc^*** being immobilized after binding to soil compartments. In some approaches, a very faint PrP***^Sc^*** specific signal was visible when analyzing aliquots of SDS solution in which the gauze bag had been washed, however, it could be shown that this positive prion signal was a result of adherent soil particles (figure 3, lane 3, arrow).

### Bioassay Experiments

**Figure 4:**
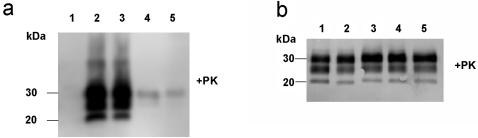
Western blot analysis of hamsters orally challenged with contaminated soil and western blot typing of PrP27-30. a) Western blot showing PrP^Sc^ in the brains of hamster orally challenged with contaminated soil samples and in the soil samples used for the bioassay. Lane 1: negative control hamster; lane 2: hamster H19 fed 12 times with aqueous extracts of soil samples from month 26 and 29; lane 3: hamster H4B fed 12 times with soil samples from month 26 and 29; lane 4: scrapie-contaminated soil (18 months); lane 5: scrapie-contaminated soil (21 months); lane 6: negative soil sample. b) Western blot typing of electrophoretic mobilities and glycosylation characteristics of PrP27-30 from different hamster-adapted TSE reference isolates, and from hamsters perorally challenged with 263K scrapie-contaminated soil. Lane 1: ME7-H scrapie agent; lane 2: hamster-adapted BSE-isolate (BSE-H); lane 3: 263K scrapie agent, lane 4: H10 (hamster 10, fed 12 times with soil samples from months 26 and 29); lane 5: H7 (hamster 7 fed 12 times with soil samples from month 26 and 29).

For a detailed risk assessment of scrapie-contaminated soil it was of major importance to analyze whether the detectable PrP^Sc^ in the soil extracts still exhibited oral infectivity after incubation times up to 29 months. Therefore, a bioassay with Syrian hamsters was performed by feeding the animals with contaminated soil or aqueous soil extracts that had been collected after soil incubation for 26 and 29 months, respectively. Hamsters fed with contaminated soil exhibited first scrapie-associated symptoms at 131±6 days [mean±SD] after the first application. The hamsters reached the terminal stage of scrapie at 162±12 days after the first feeding (tables 1 & 2). This indicates substantial amounts of persistent infectivity in soil that had been incubated for 26 and 29 months. After reaching the terminal stage of scrapie the animals were sacrificed and analyzed for the presence of PrP^Sc^ in their brains. As shown in figure 4a (lanes 2 and 3 depicting two hamsters exemplarily), the brains of all animals in this group contained high amounts of PrP^Sc^. In addition, comparison of the electrophoretic and glycosylation profiles of the brain samples from these hamsters (figure 4b, lanes 4 and 5) with those of hamsters challenged with different TSE isolates (figure 4b, ME7-H, lane 1; BSE-H, lane 2, 263K, lane 3) confirmed, that strain specific biochemical properties of the pathological prion protein were maintained in the animals fed with soil that had been incubated with 263K scrapie brain homogenate.

**Table 1:** Feeding schedule for bioassay in Syrian hamsters

Date	amount of soil or aqueous extract administered orally*	Collection time of soil sample
31/01/06	100 mg or 100 µl	month 26
07/02/06	100 mg or 100 µl	month 26
14/02/06	100 mg or 100 µl	month 26
21/02/06	100 mg or 100 µl	month 26
28/02/06	100 mg or 100 µl	month 26
08/03/06	100 mg or 100 µl	month 26
14/03/06	100 mg or 100 µl	month 26
21/03/06	50 mg or 50 µl	month 26
28/03/06	100 mg or 100 µl	month 29
06/04/06	100 mg or 100 µl	month 29
11/04/06	100 mg or 100 µl	month 29
20/04/06	100 mg or 100 µl	month 29

* mg relates to soil sample; µl relates to aqueous extract

**Table 2:** Infection rate in hamster after oral challenge

Group	No. of animals infected with scrapie	Terminal stage of scrapie after the first oral application/Mean incubation period with the respective standard deviation in days
Control	0/5*	–
Control soil	0/5*	–
Control aqueous extract	0/5*	–
Contaminated soil	12/12	162 +/− 12
Contaminated aqueous soil extract	4/11*	256 +/− 41

* In the marked groups one animal each died at the beginning of the bioassay because of digestive disorders unrelated to scrapie disease

Remarkably, not only the hamsters fed with contaminated soil but also four hamsters of the group fed with aqueous soil extracts developed terminal scrapie at 256±41 days after the first application (with incubation times of individual animals ranging from 201 to 321 days). Since this bioassay is ongoing, further animals of this group might still develop scrapie in the future.

## Discussion

The results of this research project show for the first time that the scrapie strain 263K remains persistent in soil over a period of at least 29 months and remains highly infectious after oral application to Syrian hamsters. It has to be pointed out that the key results of our time-course study on the fate of PrP^Sc^ in soil have been validated, in part by examining blinded samples, at independent laboratories.

Only a few studies have addressed the question of a persistence of prions in soil so far [24]–[26], [47], and the results from these studies are in principle in accordance with our observations. A pioneering study was published by Brown and Gajdusek in 1991 [24] showing that an aqueous extract from scrapie-contaminated soil remains infectious even after an incubation period of three years as confirmed by hamster bioassay. However, the infectivity studies were conducted by intracerebral injection and not by oral application. Furthermore, the PrP^Sc^ concentration was not analyzed in this study, so that no data are available about the proteińs absorption behavior to soil particles and about the corresponding degradation kinetics. Most recently, PrP^Sc^ has been shown to bind to soil minerals [25] but only short-time incubation experiments of maximal one week were conducted and, again, bioassays were performed by the intracerebral route.

In this study we show by Western blotting a strong decrease in the amount of extractable PrP^Sc^ over an incubation period of 29 months in soil. It is not yet clear whether this decrease resulted from a molecular degradation of PrP^Sc^ or a tighter binding to soil particles. Stronger binding of molecules to soil particles with increasing incubation time is a well-known phenomenon in soil chemistry – the so called “aging” – and influences bioavailability and re-mobilization significantly [48], [49].

Upon feeding hamsters with scrapie contaminated soil which had been incubated for over two years in outdoor lysimeters all animals developed terminal scrapie after relatively short incubation times (162 dpi). In other studies it has been well established that pure 10% (w/v) brain homogenates from 263K scrapie hamsters cause terminal scrapie in perorally challenged hamsters after mean incubation times of about 155–165 days with an attack rate of 100% [50]–[53]. This indicates that scrapie-contaminated soil may represent a potential TSE hazard for ruminants in the environment. While a considerable excretion of infectivity has to be assumed for scrapie or BSE infected sheep and CWD infected deer [40], [43], [54], it is generally acknowledged that the potential environmental contamination risk represented by BSE infected cattle is marginal, if at all present [41], [42]. On the other hand, the burial of bovine carcasses [44] might have accidentally led to a spill of BSE prions into the environment. Furthermore, the fact that even feeding of aqueous extracts from scrapie-contaminated soil induced a terminal scrapie infection in four hamsters so far suggests that surface water or groundwater from pastures of scrapie-affected flocks may provide a potential source of scrapie infectivity.

However, the relevance of the results obtained in this study for the field situation should be interpreted with some caution, since only one soil type was used and only a limited number of animals were challenged in the bioassay. Therefore, other soil types and a larger number of animals have to be tested in future studies to allow for a robust risk assessment. Furthermore the exact binding properties and degradation kinetics of PrP^Sc^ should be subject to further research. In addition, all published studies addressing the persistence of prion infectivity in soil were performed with scrapie prions while TSE agents causing BSE and especially CWD have not been analyzed so far.

An intensified monitoring of PrP^Sc ^(and possibly also prion infectivity) in the soil appears mandatory for a more precise assessment of the risks emanating for humans and animals from prions in the environment. As shown in this report, PrP^Sc^ extracted from soil can be used as a catalytically active seed in the protein misfolding cyclic amplification (PMCA) reaction. This opens a promising perspective for considerably improving the detectability of prions in the environment.

## Materials and Methods

### Biological Safety

Assays with scrapie-infected hamster brain were performed under laboratory conditions according to bio safety level 3** and in protected outdoor lysimeters, respectively.

### Extraction of Brain Material

To identify a suitable buffer for extracting prion infectivity and PrP^Sc^ from soil, German standard soil (Lufa 2.2 and Borstel) was mixed with scrapie-infected hamster brain (strain 263K) provided by the TSE-Resource-Centre, Berkshire, Great Britain. To test the efficiency of this method the following solutions and buffers - especially non-ionic and ionic detergents - have been tested:

a) sterile water, b) 1% urea in sterile water c) 1% SDS in sterile water, d) 1% Zwittergent 3-08 in sterile water, e) 1% Triton X-100 sterile water, f) 10% Na-sarcosylate in sterile water, g) RIPA-buffer (0.25% Na-deoxycholate, 0.9% NaCl, 1% NOP-40, 0.8% Tris-HCl in sterile water (Carl Roth GmbH, Karlsruhe, Germany), h) 10% NOP-40 in sterile water, (Sigma-Aldrich, Steinheim, Germany).

Initially non-infectious pork brain was mixed with German standard soil and the efficiency of PrP^C^ extraction using the above mentioned buffers was tested. In a second step, the optimal extraction procedure was verified by applying the procedure to soil samples mixed with 263K scrapie agent and monitoring the PrP^Sc^ retrieval.

### Incubation Experiments and Sampling Scheme

The outdoor experiments and all other experiments were performed using brains of terminally-diseased hamsters challenged with scrapie strain 263K.

For each approach, 1 g infectious hamster brain material was homogenized in 10 ml PBS (phosphate buffered saline) and added to 20 g of standard German sandy loom soil (Borstel). The soil/brain mixture was filled into gauze bags and buried in lysimeters filled with the same soil at a depth between 15 and 20 cm. The gauze had a mesh size of 250 µm, which enables the contact with microorganisms and meso fauna with the soil/brain mixture but avoids the contact with macro fauna derived/related organisms. To protect the gauze bags from mice, the bags were put into steel cages. At defined time points (after 0, 1, 3, 6, 12, 18, 21, 26, 29 months) the steel cages were dug out and the gauze bags as well as the surrounding soil were taken for sampling and analyzing. The analyses on the presence of residual PrP27-30 in soil samples were performed with a different number of soil bags, depending on the incubation times to be tested. For “short” incubation times of <12 months, for which detectability of residual PrP appeared most likely when the experiments were designed, samples from three different soil bags were examined. For “intermediate” incubation times of 12–18 months samples from two different bags were tested, and for “long” incubation times of >18 months one soil bag was yet available for sampling. All bags were buried in close vicinity.

### Sample Preparation and Western Blot Analysis of Soil and Hamster Brain Samples

#### Remark:

Western blot experiments were performed in independent runs at different laboratories, with each laboratory using its established techniques and procedures. The protocols are listed below. The different analytical protocols produced consistent results.

### Western Protocol I

For the protein extraction from the contaminated soil as well as from surrounding soil samples, 20 ml of a 1%-SDS-solution (SDS; Sigma-Aldrich, Steinheim, Germany) were added to 20 g of testing material in a 50 ml tube. The suspension was vigorously shaken on a horizontal shaker for approx. 1 h, followed by a centrifugation step at 5,000 rpm for 20 min. 200 µl of the clear supernatant was incubated with proteinase K (50 µg/ml; 37°C; 1 h, Carl Roth GmbH, Karlsruhe, Germany) to eliminate non-resistant proteins. After digestion, the supernatants were boiled for 5 min in Laemmli's sampling buffer (50 mM Tris (pH 6.8), 2% SDS, 10% glycerol, 50 mM ß-mercaptoethanol and 0.001% bromphenol blue) in a 1.5 ml tube and analyzed by Western blotting. The prepared samples were stored at −80°C.

For screening, Western blot samples were boiled for 5 min and separated by polyacrylamide gel electrophoresis (SDS-PAGE) by using 8–16% Tris-Glycine-SDS precast gels (i-Gels, Gradipore, LTF-Labortechnik, Wasserburg, Germany) or 4–20% precast gels (Precise Protein Gels, Perbio Science GmbH, Heidelberg, Germany) according to standard procedures as described previously [55]–[57]. After SDS-PAGE, the proteins were transferred to a polyvinylidene difluoride membrane (PVDF, Immobilon Millipore, Billerica, USA) using a semi-dry blotting system. Membranes were blocked in Superblock (Perbio Science GmbH, Heidelberg, Germany) with 0.05% Tween 20 over night at 4°C. Blots were incubated for 1 h in primary antibody solution (monoclonal anti-PrP 3F4, Chemicon International, Inc., California, dilution: 1∶3,000 in PBS and 1.5% BSA or monoclonal anti-PrP 3F4, Sigma-Aldrich, Steinheim, Germany, dilution: 1∶4,000 in PBS and 1.5% BSA or monoclonal anti-mouse 6H4, Prionics AG, Zürich, Switzerland, 1∶3,000 in PBS). After washing 5 times with PBS for 5 min and 0.05% Tween 20 (Perbio Science GmbH, Heidelberg, Germany), blots were incubated in a secondary antibody solution (horseradish peroxidase-conjugated anti-mouse, dilution 1∶500,000 Perbio Science GmbH or alkaline-phosphatase-conjugated anti-mouse, dilution 1∶5,000 Sigma-Aldrich, Steinheim, Germany) for at least 1.5 h. Membranes were washed as described before and developed with CDP-Star solution (Invitrogen GmbH, Karlsruhe, Germany) or SuperSignal West Femto Maximum Sensitivity substrate (Perbio Science GmbH, Heidelberg, Germany) for 5 to 10 min. Protein signals were visualized using Pierce film (Perbio Science GmbH, Heidelberg, Germany).

For confirmation of these results, highly sensitive Western blotting was performed in independent experiments at different laboratories using their established protocols.

### Western Protocol II

The protein extractions from contaminated soil samples were carried out by adding 2 ml 1%-SDS-solution to 2 g of testing material in a 15 ml tube. The suspension was vigorously shaken for 2 min, followed by a centrifugation step at 7,000 rpm for 10 min. 50 µl of the clear supernatant was used for proteinase K digestion (100 µg/ml; 37°C; 1 h). After digestion the supernatants were mixed with an equal volume of 2× sample loading buffer (50 mM Tris (pH 6.8), 2% SDS, 10% glycerol, 50 mM ß-mercaptoethanol and 0.001% bromphenol blue) and heated to 100°C for 5 min for PrP-Western blotting.

Sodium dodecyl sulfate-polyacrylamide gel electrophoresis (SDS-PAGE) and Western blot analyzes of samples from hamsters were performed as described elsewhere [58] with recently published modifications [39]. In short: Following SDS-PAGE, proteins were transferred to a polyvinylidene difluoride (PVDF) membrane (Immobilon, Millipore, Billerica, USA) using a semi-dry blotting system. The membrane was blocked by incubation for 30 min in TBS containing 3% (w/v) nonfat milk powder (NFMP) and 0.05% (w/v) Tween-20 (NFMP-TBST). Blots were incubated overnight in primary antibody solution (monoclonal anti-PrP antibody 3F4 [mAb 3F4] from cell culture, diluted 1∶2,000 in NFMP-TBST) at 4°C. After washing 5 times for at least 20 min with NFMP-TBST, blots were incubated in secondary antibody solution (alkaline phosphatase conjugated goat anti-mouse IgG; Dako, Glostrup, Denmark; 1∶5,000 diluted in NFMP-TBST) for 90 min at room temperature. After washing 5 times with NFMP-TBST over a total period of at least 1.5 h, the membranes were pre-incubated 2×5 min in assay-buffer (100 mM Tris, 100 mM NaCl, pH 9.5) and finally developed with CDP-star solution (Tropics, Applied Biosystems, Beford, USA) for 5 min according to the instructions of the manufacturer. PrP-signals were visualized on a X-OMAT AR (Kodak, Sigma-Aldrich, Steinheim, Germany) film. Films were exposed for 5–30 min.

For deglycosylation, 500 µl of the soil extract solution were dialyzed in PBS for 16 h in order to remove the detergents, and 20 µl of the aliquot was digested using PNGase F (New England Biolabs, Ipswich, USA) according to the instructions of the manufacturer prior to Western blotting.

### Western Protocol III

Sample preparation and extraction of the prion protein from soil samples as well as proteinase K digestion was performed as described in protocol I. After electrophoresis using 16% bis-acrylamide gels, proteins were transferred on a PVDF-membrane in a semi-dry chamber. The membranes were blocked in 5% dry fat milk in PBS (phosphate buffered saline) containing 0.1% Tween 20 (Merck, Darmstadt, Germany) (PBS-T) for 30 min and subsequently incubated with the PrP-specific monoclonal detection antibody 3F4 (Chemicon International, Inc., California) in a dilution of 1∶3,000 in 5% dry fat milk in PBS-T for 1 h 30 min. The membranes were washed 3 times for 10 min with PBS-T and then incubated with a secondary antibody bound to alkaline phosphatase in a dilution of 1∶2,000 in PBS-T (goat-anti-mouse-AP, Dianova, Hamburg, Germany) for 1 h. After again washing three times for 10 min, the membranes were incubated 2 times for 2 min in assay buffer containing 200 mM Tris-HCl and 10 mM MgCl_2_ (pH 9.8). Finally, the chemiluminescence substrate CDP-Star (Tropix, Bedford, USA) was applied and incubated on the membrane for 5 min before the light signals were detected in a camera using the analysis software Quantity One (Bio-Rad, Munich, Germany).

### Bioassay in Syrian hamsters

Forty two female Syrian hamsters, approx. 90 days old, were obtained from Charles River Laboratories, Germany. They were handled according to the regulations of the local authorities (Bezirksregierung Arnsberg, reference number 50.8735.1 Nr. 108/1) in a biosafety Level 3 containment facility.

12 animals were fed weekly over a period of 12 weeks; 11 times with 100 mg soil/brain mixture each and one time with 50 mg soil/brain mixture taken from the outdoor lysimeters after an incubation period of 26 and 29 months (table 1). Oral application was achieved by mixing the soil (1,150 mg in total) with commercial hamster feed (ssniff, Soest, Germany).

To analyze the remaining infectivity in the aqueous extract, 10 g contaminated soil was mixed with 10 ml water and was vigorously shaken on a horizontal shaker for approx. 1 h. The mixture was centrifuged with 5,000 rpm for 5 min and the supernatant was used for the bioassay. Oral application was performed weekly over a period of 12 weeks (1,150 µl in total) by mixing the extract with commercial hamster feed (ssniff, Soest, Germany). For this purpose, 12 additional animals were fed 11 times with 100 µl and 1 time with 50 µl aqueous extract from the soil/brain mixture taken from the outdoor lysimeters after an incubation period of 26 and 29 months.

The hamsters of both groups were monitored at least twice a week for the development of clinical signs of scrapie. Hamsters diseased with 263K scrapie showed head bobbing, ataxia of gait and generalized tremor. Such animals were frequently and persistently in motion, easily irritated by noise and touch, upon which they often twitch, and had difficulties maintaining balance and rising from a supine position. These clinical symptoms of hamsters are entirely consistent with those previously reported for the 263K scrapie agent [59]. At the occurrence of first neurological symptoms, the animals were observed daily and at appropriate end-points, clinically affected hamsters were killed. Brains from these hamsters were examined and analyzed for the presence of PrP^Sc^.

As a control, six animals were fed with non-contaminated standard soil and 6 animals were fed with aqueous extract from non-contaminated standard soil over a period of 12 weeks (1,150 mg or 1,150 µl in total). Additional 6 hamsters were fed only with commercial hamster feed (ssniff, Soest, Germany).

### PMCA-Reaction

The PMCA method was carried out as reported previously [45], [46] with modifications: In brief, normal hamster brains were homogenized at 10% (w/v) in PBS containing complete protease inhibitor cocktail (Boehringer-Ingelheim, Mannheim, Germany), 4 mM EDTA and 1% Triton-X-100. The homogenate was briefly centrifuged at 2,000 g and the supernatant was used as the PrP^C^ source. Amplification was performed using an automatic ultrasonification apparatus (Sonicator 300 from Misonix, New York, USA) with a water-tank.

The PrP^C^ substrate was mixed with 1/10 volume of the soil extract previously dialyzed in PBS, resulting in a total volume of 100 µl. PMCA amplification was performed by 40 cycles of sonication (40 sec. each) followed by incubation at 37°C for 1 h in the water-tank. The amplified product of the first round of amplification was diluted 1∶5 with normal brain homogenate and the second round was performed. This process was repeated 4 times to obtain 160 cycles of PMCA. From each PMCA-amplification round, aliquots of 50 µl were taken and digested with proteinase K (180 µg/ml) for 1 h at 56°C. Finally, the same volume of 2× sample buffer was added and heated for 10 min at 100°C prior to SDS-PAGE.

### Western Blot Typing of PrP27-30

Sodium dodecyl sulfate-polyacrylamide gel electrophoresis (SDS-PAGE) and Western blot analyzes of samples from hamsters for characterization of the PrP^Sc^ glycosylation and migration pattern were performed as described elsewhere [59]. In short: 50 µl of 10% (w/v) brain homogenates in TBS (pH 7.4) were mixed with 5 µl of 13% (w/v) sarcosyl and 10 µl proteinase K stock solution (1 mg/ml; Roche, Mannheim, Germany) and digested for 60 min at 37°C. The digestion was stopped by adding 435 µl 2× sample buffer, i.e. 4% (w/v) SDS, 10% (v/v) 2-mercaptoethanol in 120 mM Tris-HCl, pH 6.8, containing 20% (w/v) glycerol and 0.05% (w/v) bromophenol blue, and boiling for 5 min. 5 µl of the solution (corresponding to 5×10^−5^ grams of homogenized brain tissue) were separated in a 15% SDS-PAGE or in Tris-glycine gels (Novex, Invitrogen, Carlsbad, USA) and subsequently blotted onto polyvinylidene difluoride (PVDF) membranes (Millipore, Billerica, USA) using the semi-dry method (Fast-Blot; Biometra, Göttingen, Germany). The PVDF-membranes were blocked with 5% low-fat milk in TBS for 30 min and incubated overnight at 4°C with MAb 3F4 (1∶2,000) in 3% bovine serum albumin (BSA) in TBS. After washing in TBS and incubation for 60 min at room temperature with the secondary antibody [biotinylated goat anti-mouse IgG (1∶2,000) in 3% BSA in TBS], a biotin-streptavidin kit (Dako, Glostrup, Denmark) for signal enhancement was applied for 30 min at room temperature. After washing the membranes in TBS, antibody binding to PrP was visualized using a mixture of nitroblue-tetrazoleum (NBT; Sigma-Aldrich, Steinheim, Germany) and 5-bromo-4-chloro-3-indolylphosphate (BCIP; Sigma-Aldrich, Steinheim, Germany) as substrate.

## References

[1] Prusiner SB (1998). Prions.. Proceedings of the National Academy of Sciences USA..

[2] Miller MW, Williams ES, Sigurdson CJ (2003). Prion disease: horizontal prion transmission in mule deer. Other animal prion diseases.. Nature.

[3] Williams ES, Miller MW (2002). Chronic wasting disease in deer and elk in North America.. Rev Sci Tech.

[4] Williams ES (2005). Chronic wasting disease.. Vet Pathol.

[5] Hoinville LJ (1996). A review of the epidemiology of scrapie in sheep.. Rev Sci Tech.

[6] Greig JR (1940). Scrapie: Observation on the transmission of the disease by mediate contact.. Vet J.

[7] Pállson PA (1979). Rida in Iceland an dits epidemology. In Prusiner SB, Hadllow WJ, Slow transmissible diseases of the nervous system.. Academic Press.

[8] Miller MW, Williams ES (2003). Prion disease: horizontal prion transmission in mule deer.. Nature.

[9] Kim TY, Shon HJ, Joo YS, Mun UK, Kang KS, Lee YS (2005). Additional cases of Chronic Wasting Disease in imported deer in Korea.. J Vet Med Sci,.

[10] Sohn HJ, Kim JH, Choi KS, Nah JJ, Joo YS (2002). A case of chronic wasting disease in an elk imported to Korea from Canada.. J Vet Med Sci.

[11] Miller MW, Williams ES, Hobbs NT, Wolfe LL (2004). Environmental sources of prion transmission in mule deer.. Emerg Infect Dis.

[12] Miller MW, Wild MA, Williams ES (1998). Epidemiology of chronic wasting disease in captive Rocky Mountain elk.. J Wildl Dis.

[13] Williams ES, Young S (1992). Spongiform encephalopathies in Cervidae.. Rev Sci Tech.

[14] Hadlow WJ, Kennedy RC, Race RE (1982). Natural infection of Suffolk sheep with scrapie virus.. J Infect Dis.

[15] Mathiason CK, Powers JG, Dahmes SJ, Osborn DA, Miller KV (2006). Infectious prions in the saliva and blood of deer with chronic wasting disease.. Science.

[16] Post K, Riesner D, Walldorf V, Mehlhorn H (1999). Fly larvae and pupae as vectors for scrapie.. Lancet.

[17] Lupi O (2003). Could ectoparasites act as vectors for prion diseases?. Int J Dermatol.

[18] Lupi O (2005). Risk analysis of ectoparasites acting as vectors for chronic wasting disease.. Med Hypotheses.

[19] Wisniewski HM, Sigurdarson S, Rubenstein R, Kascsak RJ, Carp RI (1996). Mites as vectors for scrapie.. Lancet.

[20] Purdey M (1998). High-dose exposure to systemic phosmet insecticide modifies the phosphatidylinositol anchor on the prion protein: the origins of new variant transmissible spongiform encephalopathies?. Med Hypotheses.

[21] Purdey M (2000). Ecosystems supporting clusters of sporadic TSEs demonstrate excesses of the radical-generating divalent cation manganese and deficiencies of antioxidant co factors Cu, Se, Fe, Zn. Does a foreign cation substitution at prion protein's Cu domain initiate TSE?. Med Hypotheses.

[22] Gudmundsdottir KB, Sigurdarson S, Kristinsson J, Eiriksson T, Johannesson T (2006). Iron and iron/manganese ratio in forage from Icelandic sheep farms: relation to scrapie.. Acta Vet Scand.

[23] Sigurdsson B (1954). Rida, a chronic encephalitis of sheep. With general remarks on infectious which develop slowly and some of their special characteristics.. British Veterinary Journal.

[24] Brown P, Gajdusek DC (1991). Survival of scrapie virus after 3 years' interment.. Lancet.

[25] Johnson CJ, Phillips KE, Schramm PT, McKenzie D, Aiken JM (2006). Prions adhere to soil minerals and remain infectious.. PLoS Pathog.

[26] Rigou P, Rezaei H, Grosclaude J, Staunton S, Quiquampoix H (2006). Fate of prions in soil: adsorption and extraction by electroelution of recombinant ovine prion protein from montmorillonite and natural soils.. Environ Sci Technol.

[27] Gibbons RA, Hunter GD (1967). Nature of the scrapie agent.. Nature.

[28] Bolton DC, McKinley MP, Prusiner SB (1982). Identification of a protein that purifies with the scrapie prion.. Science.

[29] McKinley MP, Bolton DC, Prusiner SB (1983). A protease-resistant protein is a structural component of the scrapie prion.. Cell.

[30] Diringer H, Gelderblom H, Hilmert H, Ozel M, Edelbluth C (1983). Scrapie infectivity, fibrils and low molecular weight protein.. Nature.

[31] Alper T, Haig DA, Clarke MC (1966). The exceptionally small size of the scrapie agent.. Biochem Biophys Res Commun.

[32] Taylor DM (1993). Inactivation of SE agents.. Br Med Bull.

[33] Taylor DM (1991). Inactivation of the unconventional agents of scrapie, bovine spongiform encephalopathy and Creutzfeldt-Jakob disease.. The Journal of Hospital Infection.

[34] Taylor DM (2000). Inactivation of transmissible degenerative encephalopathy agents: A review.. Vet J.

[35] Taylor DM, Fernie K, McConnell I (1997). Inactivation of the 22A strain of scrapie agent by autoclaving in sodium hydroxide.. Veterinary Microbiology.

[36] Taylor DM, Fernie K (1996). Exposure to autoclaving or sodium hydroxide extends the dose-response curve of the 263K strain of scrapie agent in hamsters.. The Journal of General Virology.

[37] Taylor DM, Fernie K, McConnell I, Steele PJ (1999). Survival of scrapie agent after exposure to sodium dodecyl sulphate and heat.. Veterinary Microbiology.

[38] Shaked GM, Shaked Y, Kariv-Inbal Z, Halimi M, Avraham I (2001). protease-resistant prion protein isoform is present in urine of animals and humans affected with prion diseases.. J Biol Chem.

[39] Thomzig A, Kratzel C, Lenz G, Kruger D, Beekes M (2003). Widespread PrPSc accumulation in muscles of hamsters orally infected with scrapie.. EMBO reports.

[40] Miller MW, Williams ES (2004). Chronic wasting disease of cervids.. Curr Top Microbiol Immunol.

[41] Bradley R, Wilesmith JW (1993). Epidemiology and control of bovine spongiform encephalopathy (BSE).. Br Med Bull.

[42] Anderson RM, Donnelly CA, Ferguson NM, Woolhouse ME, Watt CJ (1996). Transmission dynamics and epidemiology of BSE in British cattle.. Nature.

[43] Schreuder BE, Somerville RA (2003). Bovine spongiform encephalopathy in sheep?. Rev Sci Tech.

[44] Scudamore JM, Harris DM (2002). Control of foot and mouth disease: lessons from the experience of the outbreak in Great Britain in 2001.. Rev Sci Tech.

[45] Saborio GP, Permanne B, Soto C (2001). Sensitive detection of pathological prion protein by cyclic amplification of protein misfolding.. Nature.

[46] Saa P, Castilla J, Soto C (2006). Ultra-efficient replication of infectious prions by automated protein misfolding cyclic amplification.. J Biol Chem.

[47] Cindy MCooke, Joanne Rodger, Allister Smith, Karen Fernie, George Shaw, Robert ASomerville (2007). Fate of Prions in Soil: Detergent Extraction of PrP from SoilsEnviron.. Sci. Technol..

[48] Hatzinger PB, Alexander M (1995). Effect of aging of chemicals in soil on their biodegradability and extractability.. Environ. sci. technol.

[49] Scow KM, Fan S, Johnson C, Ma GM (1995). Biodegradation of sorbed chemicals in soil.. Environ Health Perspect.

[50] Beekes M, McBride PA, Baldauf E (1998). Cerebral targeting indicates vagal spread of infection in hamsters fed with scrapie.. J Gen Virol.

[51] McBride PA, Beekes M (1999). Pathological PrP is abundant in sympathetic and sensory ganglia of hamsters fed with scrapie.. Neurosci Lett.

[52] McBride PA, Schulz-Schaeffer WJ, Donaldson M, Bruce M, Diringer H, Kretzschmar HA, Beekes M (2001). Early spread of scrapie from the gastrointestinal tract to the central nervous system involves autonomic fibers of the splanchnic and vagus nerves.. J Virol.

[53] Thomzig A, Schulz-Schaeffer W, Kratzel C, Mai J, Beekes M (2004). Preclinical deposition of pathological prion protein PrPSc in muscles of hamsters orally exposed to scrapie.. J Clin Invest.

[54] Foster JD, McKelvey WA, Mylne MJ, Williams A, Hunter N (1992). Studies on maternal transmission of scrapie in sheep by embryo transfer.. Vet Rec.

[55] Seidel B, Braeg S, Adler G, Wedlich D, Menke A (2004). E- and N-cadherin differ with respect to their associated p120ctn isoforms and their ability to suppress invasive growth in pancreatic cancer cells.. Oncogene.

[56] Menke A, Vogelmann R, Seidel B, Lutz MP, Adler G (2001). E-cadherin gene expression by collagen type I and type III in pancreatic cancer cell lines.. Cancer Research.

[57] Giehl K, Seidel B, Gierschik P, Adler G, Menke A (2000). TGFbeta1 represses proliferation of pancreatic carcinoma cells which correlates with Smad4-independent inhibition of ERK activation.. Oncogene.

[58] Beekes M, Baldauf E, Diringer H (1996). Sequential appearance and accumulation of pathognomonic markers in the central nervous system of hamsters orally infected with scrapie.. J Gen Virol.

[59] Thomzig A, Spassov S, Friedrich M, Naumann D, Beekes M (2004). Discriminating scrapie and bovine spongiform encephalopathy isolates by infrared spectroscopy of pathological prion protein.. J Biol Chem.

